# The effect of detox solution on color stability, roughness, and microhardness of monochromatic universal composite resins

**DOI:** 10.1186/s12903-024-04587-x

**Published:** 2024-07-13

**Authors:** Merve Nezir, Cansu Dağdelen Ahisha, Suat Özcan, Mine Betül Üçtaşli

**Affiliations:** Faculty of Dentistry, Department of Restorative Dentistry, Gazi University, Bişkek St. 1. St. Number: 8 Emek, Ankara, Turkey

**Keywords:** Detox solution, Discoloration, Microhardness, Universal composite resin, Surface roughness

## Abstract

**Background:**

Currently, the advantages of monochromatic universal composite resin restorative materials have increased their use in dentistry. Accordingly, the optical, surface and mechanical properties of these materials have become more important. This study aimed to evaluate the effect of detox solution on discoloration, surface roughness (SR), and microhardness of different monochromatic universal composite resins (Omnichroma [O], Zenchroma [Z], Vittra [V], and Charisma Diamond One [CDO]). Another aim of this study was to evaluate the monomer conversion degree (DC) of the materials.

**Methods:**

A total of 80 specimens were prepared to evaluate the materials (*n* = 10). After the initial measurements, the specimens were immersed in a red detox solution for 21 days. Statistical data analysis was performed using one-way ANOVA and Tukey’s multiple comparisons.

**Results:**

The ∆E values of Z were highest on the 21st day. There was an increase in the SR values of the materials immersed in the detox solution. On the 21st day, top surface microhardness of O was lower than the other materials. There was no statistically significant difference at DC values among material groups.

**Conclusions:**

The use of detox solutions for a commercially recommended period of 21 days is suggested. However, this usage period can cause discoloration in restorative materials. Furthermore, especially in the initial one-week period, detox solution may have a negative impact on the microhardness of the materials. In light of all these data, we recommend the cautious use of detox solutions to prevent adverse effects on restorative materials.

## Background

In dentistry, color selection and shades resembling dental tissues are essential, challenging, and clinician-dependent. When selecting restorative materials in the clinic, minimizing color selection, simplifying protocols, and reducing chairside time are desired. Universal composite resins are materials with a monochromatic that mimic the aesthetic features of dental tissues. Despite being monochromatic, these composite resins are claimed to be compatible with various tooth shades. Furthermore, manufacturers argue that these composite resins possess an advanced “color adjustment potential (CAP),” which defines and quantifies the interaction between perceptual and physical components obtained through visual and instrumental color measurements [1].

Universal composite resins are materials with good polishability, aiming to achieve both the strength required to restore posterior teeth and the high gloss needed to mimic enamel in anterior teeth. Therefore, for a successful esthetic restoration of tooth color, the restorative material should exhibit good mechanical and physical properties and provide adequate esthetic characteristics and color compatibility [2]. According to manufacturers, these composite resins’ primary advantage is their advanced color adjustment potential (CAP). These materials have a universal opacity and are recommended to be used as a monochromatic layer that can match different tooth colors. Monochromatic universal composite resins have recently been developed to match all Vita Classical shades from A1 to D4 [3]. The frequent consumption of food and beverages by modern societies can lead to absorption and adsorption on the surfaces of composite resin restorations. Particularly, acidic beverages can degrade the surface of restorative materials, leading to the breakdown of the organic matrix structure. Consequently, the wear resistance, surface hardness, and roughness of composite resins are adversely affected. Discolorations can also occur due to the penetration of color pigments from food and beverages into porous areas resulting from degradation of restorative material surfaces. Discoloration of composite resin restorations is a significant reason for restoration replacement [4].

Smooth surfaces reduce gum irritation, plaque accumulation, and, as a result, recurrent caries and potential discolorations in restorations [5]. Surface roughness is generally associated with the particle size of composite resin fillers; composite resins with smaller particle sizes tend to have smoother surfaces [6]. Besides the properties of restorative materials, external factors such as pH changes can also influence surface roughness.

The color stability of composite resins is associated with material characteristics, cavity type, characteristics of the staining environment, staining time, light polymerization properties, layer thickness, and surface treatment. Color stability of these materials is often tested in vitro by immersing material specimens in staining beverages that mimic various clinical conditions, such as coffee, tea, cola, orange juice, smoothies, and detox solutions [7].

Having adequate surface hardness values is also important for restorative materials used in dentistry. Restorative materials used in dentistry are exposed to various factors in the intraoral environment, such as pH, occlusal forces, and temperature changes. Their ability to resist these effects can be achieved through high surface hardness values. Hardness plays a role in enhancing the material’s mechanical strength, scratch resistance, and wear resistance, as well as in maintaining its shape against forces. Therefore, surface hardness evaluation is an effective and valid method for estimating the clinical success of the material [8].

Composite resins have three main components: resin matrix, inorganic fillers, and silane. Additionally, pigments, inhibitors, and initiators are present in the structure. Cross-linking reactions begin with the initiator system’s effect, and carbon-carbon double bonds transform into carbon-carbon single bonds, forming polymers. The percentage of double bonds that transform into polymerizable single bonds is expressed as the monomer conversion degree (DC). The DC of resin composites used in dentistry ranges from 50 to 80%, indicating that 20–50% of the double bonds do not react. High DC values are associated with high polymerization shrinkage. In contrast, low DC values are related to low mechanical properties, color stability, and biocompatibility [9].

Temperature changes, mechanical and chemical interactions occurring in the oral environment, such as contact with acidic and pigmented foods, can have undesirable effects on dental restoration materials’ color stability and structure. Acidic foods are reported to have effects such as altering the surface roughness and decreasing microhardness in composite resins [10]. However, individuals’ increased interest in healthy food and beverage consumption has recently been observed. The use of smoothies, which consist of fruit beverages, is quite common. These beverages contain vitamins, antioxidants, polyphenols, and fibers. However, these beverages can potentially damage restorative material surfaces due to their acidic content [4]. In addition, the acids and colorants contained in these beverages can cause negative effects such as discoloration, which may affect the aesthetic appearance of the restorations [11].

The aim of this study was to evaluate the effect of four different monochromatic universal composite resin immersion in detox solution on the discoloration, surface roughness, and upper/bottom surface microhardness values of the materials. Another aim of this study was to evaluate the monomer conversion degree of the materials.

## Methods

### Specimen preparation

A total of 80 specimens, 20 specimens from each material, were prepared for the study. The prepared material specimens were divided into two subgroups (*n* = 10), one group for color change and surface roughness testing, one group for microhardness test and monomer conversion degree calculation.

The materials used in the study and their contents are shown in Table 1.

**Table 1:** Characteristics of the composition of materials used in the study

Material	Composition	Filler Ratio (Wt %)	Particle Size	Manufacturer
Omnichroma	Supra-nano spherical filler (260 nm spherical SiO_2_-ZrO_2_) 1.6 Bis-GMA, UDMA, TEGDMA	79%	2.6 μm	Tokuyama, Yamaguchi Prefecture, Japan
Charisma Diamond One	Advanced TCD matrix, BPA free	75%	5 –20 μm	Kulzer, Hanau, Germany
Vittra Unique	UDMA, TEGDMA, photoinitiator compound (APS), Zr, Si, BPA free/72–82% by weight and 52–60% by volume	67%	0.2 μm	FGM, Joinville, Brazil
Zenchroma	Glass powder, diurethane dimethacrylate, silicon dioxide, Bis-GMA, butanediol-dimethacrylate	75%	0.005 –3.0 μm	President Dental, Germany

*Abbreviations: SiO_*2*_: *Silicon dioxide*,* ZrO*_*2*_: *Zirconium dioxide*,* Bis-GMA: Bisphenol A-glycidyl methacrylate*,* UDMA: Urethane dimethacrylate*,* TEGDMA: Triethylene glycol dimethacrylate*,* BPA: Bisphenol A*,* Zr: Zirconium*,* Si: Silicon*,* µm: micrometer.*

A total of 80 disc-shaped specimens with the dimensions 7 mm x 2 mm were prepared for evaluating discoloration, surface roughness, microhardness from the top and bottom surfaces, and monomer conversion degree. A both-sided open plexiglass mold was positioned over a 1 mm thick glass slide and a mylar strip, filled with composite resin in a single increment, then the mylar strip and glass slide were placed on the plexiglass mold. A slight pressure of 5–10 N was applied to the glass slide. This pressure served two purposes: to remove any excess composite material from the mold and to ensure standardization of the specimen thickness and the distance between the specimens and the light curing tip. After that, the composite resin polymerized with a light emitting diode (LED) light curing unit (D-Light Pro, GC Europe N.V., Leuven, Belgium) in High Power [HP] mode with an intensity of 1400 mW/cm^2^ for 20 s. Following the light curing process, the specimens were polished using aluminum-oxide-coated discs (Sof-Lex, 3 M ESPE, St. Paul, MN, USA) with a slow-speed handpiece, applying each disk (coarse, medium, fine, and ultrafine) for 10 s. The specimens were kept in distilled water in an oven at 37 °C to simulate the oral environment during the experiment, except for the detox circulation application.

### Detox circulation

Monochromatic universal composite resin restorative materials were immersed in a red detox solution with a pH of 4.04 (Table 2). The application period was determined based on the duration (21 days) and frequency (simulating three daily meals, exposure time; 5 min) of detox application in daily life.

**Table 2:** Characteristics of the detox solution used in the study

Solution	Manufacturer	Contents	pH
Detox Solution	Organik Smoothie Passion Red, Elite Naturel, Ankara, Turkey	Organic watermelon juice (20%), organic strawberry puree (20%), organic banana puree (15%), organic apple puree (15%), organic pear puree (12%), organic black mulberry puree (12%), organic red beet juice (3%), organic black carrot juice (3%).	4.04

### Measurement of discoloration

The color measurement of the specimens was conducted according to the CIE L*a*b* scale using a spectrophotometer (VITA EasyShade, VITA Zahnfabrik, Bad Sackingen, Germany) after 24 h as a baseline before immersion, 7th and 21st days after immersion on a white background under standardized conditions and lighting. Before each color measurement, the specimens were washed with distilled water and dried with absorbent paper to remove detox solution residues on the specimen surfaces. All color measurements were made in the same place and at the same time of the day. Natural daylight was used when the measurements were performed. Measurements were repeated three times from the middle of each specimen. Measurements were carried out at three different points from the middle of the specimen surface. L*, a*, and b* values were recorded for each specimen after 24 h (baseline) and on the 7th and 21st days. The color difference (ΔE) between 24 h/ 7 day and 24 h/ 21 day for each specimen was calculated using the following equation:

ΔE*=[(∆L)^2^+(∆a)^2^+(∆b)^2^]^1/2^.

ΔE*=[(L*_1_-L*_0_)^2^+(a*_1_-a*_0_)^2^+(b*_1_-b*_0_)^2^]^1/2^.

### Measurement of surface roughness

After 24 h of specimen preparation and on the 7th and 21st days, the surface roughness of the specimens was measured using a profilometer (Surftest SJ-301-Mitutoyo, Illinois, USA) within an area of 100*100 µm² in three different planes. The mean of the measurement values obtained was recorded as the surface roughness value for that specimen.

### Measurement of top-bottom surface microhardness

The microhardness values of the prepared specimens’ top and bottom surfaces were determined using a Vicker’s Hardness Measurement Device (HMV-700 Microhardness Tester, Shimadzu, Japan). A load of 100 g, equivalent to 980.7 mN, was applied for 10 s during testing. The resulting rhombus-shaped impressions were marked under x40 magnification, and Vicker’s hardness values were recorded. Measurements were conducted at three different points for each specimen. The mean of the obtained measurement values was saved as the top-bottom surface microhardness value for that specimen.

### Calculation of monomer conversion degree (DC)

It has been reported that surface microhardness indicates the monomer conversion degree (DC) and exhibits a strong correlation with infrared spectroscopy. The evaluation of DC relies on surface microhardness ratios ranging between 80% and 90%. When the ratio of bottom surface microhardness to top surface microhardness of a restorative material exceeds 80%, it signifies sufficient polymerization [12]. In this study, this ratio was used to calculate the monomer conversion degree:

$$\begin{array}{l}\:\text{D}\text{e}\text{g}\text{r}\text{e}\text{e}\:\text{o}\text{f}\:\text{m}\text{o}\text{n}\text{o}\text{m}\text{e}\text{r}\:\text{c}\text{o}\text{n}\text{v}\text{e}\text{r}\text{s}\text{i}\text{o}\text{n}\\=\frac{\text{B}\text{o}\text{t}\text{t}\text{o}\text{m}\:\text{s}\text{u}\text{r}\text{f}\text{a}\text{c}\text{e}\:\text{m}\text{i}\text{c}\text{r}\text{o}\text{h}\text{a}\text{r}\text{d}\text{n}\text{e}\text{s}\text{s}\:\text{v}\text{a}\text{l}\text{u}\text{e}}{\text{U}\text{p}\text{p}\text{e}\text{r}\:\text{s}\text{u}\text{r}\text{f}\text{a}\text{c}\text{e}\:\text{m}\text{i}\text{c}\text{r}\text{o}\text{h}\text{a}\text{r}\text{d}\text{n}\text{e}\text{s}\text{s}\:\text{v}\text{a}\text{l}\text{u}\text{e}}\times\:100\end{array}$$

### Statistical analysis

IBM SPSS Statistics 22 software was used in the statistical analysis of the data obtained.

In the evaluation of color change and surface roughness data; The normality of the parameters was evaluated using the Shapiro-Wilk test and it was revealed that the parameters exhibited normal distribution. One-way analysis of variance (ANOVA) was used for comparison between groups. Tukey Honest Significant Difference (HSD) test was used to determine the source of differences.

In the evaluation of microhardness and monomer conversion degree data; The normality of the parameters was evaluated using the Shapiro-Wilk test and it was revealed that the parameters were not normally distributed. The Kruskal-Wallis test was used for comparisons between groups, and the Dunn test was used to determine the group responsible for the differences. Within-group comparisons were made using the Friedman Test (post hoc Wilcoxon signed-rank test). Significance was determined at *p* < 0.05.

## Results

### Discoloration results

**Fig. 1:**
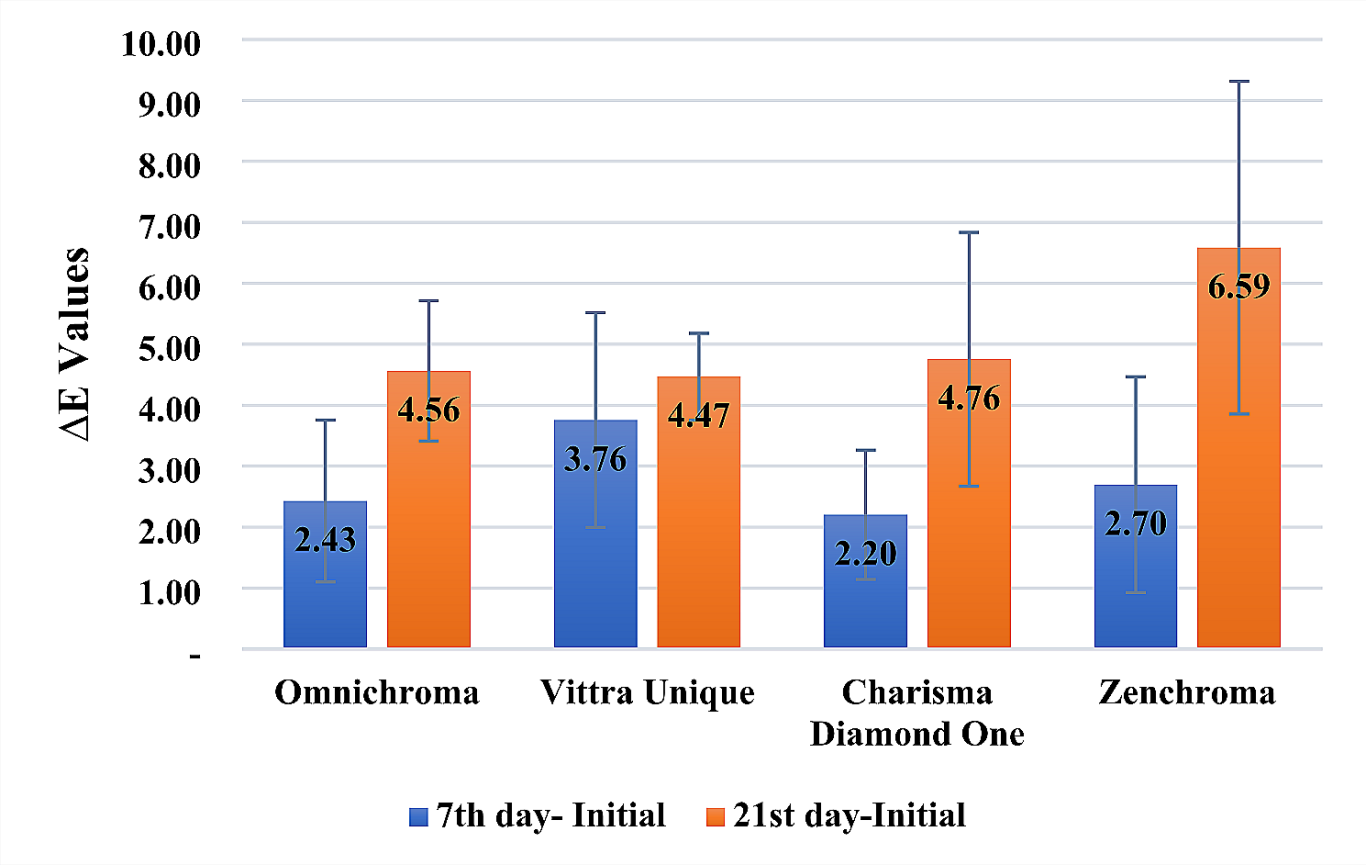
Evaluation of groups in terms of ∆E

As shown in Fig. 1, no statistically significant difference was detected among the tested monochromatic universal composite resin restorative materials regarding color measurements of specimens immersion in the detox solution for 7 days (*p* > 0.05), and all ∆E values were found within the clinically acceptable range according to the O’Brien clinical color matching table (Table 3). However, when the storage period in the detox solution was extended to 21 days, ∆E values exceeding 3.3 were observed. These results indicate that the discoloration is clinically unacceptable. In the color measurements conducted after 21 days, Zenchroma exhibited higher ∆E values compared to the other tested restorative materials (*p* > 0.05). Among the monochromatic universal composite resin restorative materials, Vittra Unique displayed a notable discoloration in the initial and 7th day evaluations, but it was regarded as the most stable restorative material concerning discoloration in the 21st day assessments.

**Table 3:** O’Brien Clinical Color Stability [29]

∆E Value	Meaning
0	Perfect
0.5–1.5	Very good
1–2	Good
2-3.5	Clinically acceptable
3.5>	Incompatible

### Surface roughness results

**Fig. 2:**
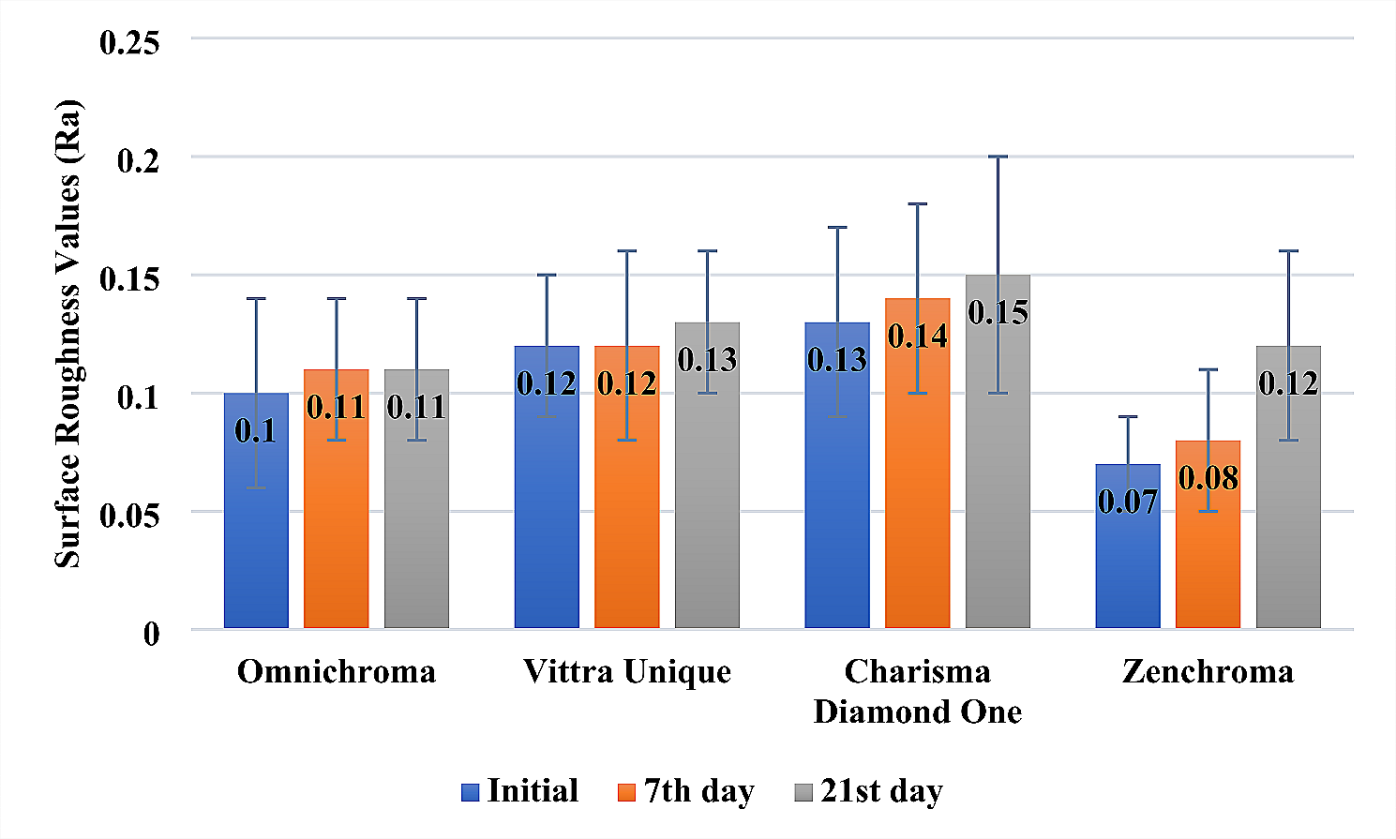
Evaluation of groups in terms of surface roughness

According to Fig. 2, although there was no statistically significant finding in the surface roughness values when compared to the baseline after immersion in the detox solution in all monochromatic universal composite resin restorative materials evaluated, an increase was observed. Among the assessed restorative materials, Zenchroma exhibited the most significant alteration in surface roughness.

### Microhardness results

There was no statistically significant difference on the initial top surface microhardness among material groups (*p* > 0.05). However, statistically significant differences were observed on top surface microhardness at day 7 (*p* < 0.05). Omnichroma exhibited significantly lower top surface microhardness than Charisma Diamond One and Zenchroma at day 7 (*p* < 0.05). Vittra Unique had significantly lower top surface microhardness than Charisma Diamond One and Zenchroma at day 7 (*p* < 0.05). Statistically significant differences were observed on top surface microhardness at day 21 (*p* < 0.05). Omnichroma had significantly lower top surface microhardness than Vittra Unique, Charisma Diamond One, and Zenchroma at day 21 (*p* < 0.05). Vittra Unique had significantly lower top surface microhardness than Charisma Diamond One and Zenchroma at day 21 (*p* < 0.05). Within the Omnichroma group, statistically significant differences on top surface microhardness were observed among the initial, day 7, and day 21 values (*p* < 0.05). The decreases on top surface microhardness values at day 7 and day 21 compared to the initial values were statistically significant (*p* < 0.05). There was no significant change on top surface microhardness from day 7 to day 21 (*p* > 0.05). In the Vittra Unique, Charisma Diamond One, and Zenchroma groups, there were no statistically significant differences on top surface microhardness among the initial, day 7, and day 21 values (*p* > 0.05) (Table 4).

**Table 4:** Evaluation of groups in terms of top Surface Microhardness

Top Surface Microhardness Values	Omnichroma	Vittra Unique	Charisma Diamond One	Zenchroma	
	Mean ± Sd	Mean ± Sd	Mean ± Sd	Mean ± Sd	^1^ *p*
Initial	98,55 ± 12,49	92,84 ± 7,76	148,72 ± 34,88	100,73 ± 14,8	0,101
7th day	75,36 ± 14,96	110,57 ± 38,57	102,1 ± 10,1	110,12 ± 17,76	0,042*
21st day	71,76 ± 7,78	92,86 ± 8,13	89,26 ± 6,22	91,42 ± 4,32	0,015*
^2^p	0,022*	0,829	0,074	0,022*	
*Initial – 7th day* ^*3*^ *p*	*0*,043*	*0*,*686*	*0*,*073*	*0*,043*	
*Initial – 21st day* ^*3*^ *p*	*0*,043*	*0*,*686*	*0*,*080*	*0*,*225*	
*7th day – 21st day* ^*3*^ *p*	*0*,*686*	*0*,*500*	*0*,*138*	*0*,043*	

^*1*^ *Kruskal Wallis Test* ^*2*^ *Friedman Test* ^*3*^ Wilcoxon sign test *p < 0.05

There was no statistically significant difference on initial bottom surface microhardness among material groups (*p* > 0.05). However, statistically significant differences were observed on bottom surface microhardness at day 7 (*p* < 0.05). Omnichroma had significantly lower bottom surface microhardness than Charisma Diamond One and Zenchroma at day 7 (*p* < 0.05). Vittra Unique had significantly lower bottom surface microhardness than Charisma Diamond One and Zenchroma at day 7 (*p* < 0.05). Statistically significant differences were observed on bottom surface microhardness at day 21 (*p* < 0.05). Omnichroma had significantly lower bottom surface microhardness than Vittra Unique, Charisma Diamond One, and Zenchroma at day 21 (*p* < 0.05). Vittra Unique had significantly lower bottom surface microhardness than Charisma Diamond One and Zenchroma at day 21 (*p* < 0.05). There were no statistically significant differences at the initial, day 7, and day 21 bottom surface microhardness values within each material group (*p* > 0.05) (Table 5).

**Table 5:** Evaluation of groups in terms of bottom surface microhardness

Bottom Surface Microhardness Values	Omnichroma	Vittra Unique	Charisma Diamond One	Zenchroma	
	Mean ± Sd	Mean ± Sd	Mean ± Sd	Mean ± Sd	^1^ *p*
Initial	75,46 ± 24,46	70,34 ± 10,37	104,38 ± 33,44	81,61 ± 8,58	0,066
7th day	66,1 ± 4,84	72,25 ± 10,74	102,17 ± 32,69	88,15 ± 8,09	0,006*
21st day	68,61 ± 4,73	68,22 ± 7,28	82,79 ± 5,41	80,08 ± 7,95	0,010*
^2^p	0,819	0,819	0,091	0,091	
*Initial – 7th day* ^*3*^ *p*	*0*,*686*	*0*,*686*	*0*,*686*	*0*,*225*	
*Initial – 21st day* ^*3*^ *p*	*0*,*893*	*0*,*500*	*0*,*138*	*0*,*893*	
*7th day – 21st day* ^*3*^ *p*	*0*,*345*	*0*,*225*	*0*,*063*	*0*,*063*	

^*1*^ *Kruskal Wallis Test* ^*2*^ *Friedman Test* ^*3*^ *Wilcoxon sign test*

*p < 0.05

### Monomer conversion degree results

There was no statistically significant difference at DC values among material groups (*p* > 0.05) (Table 6).

**Table 6:** Evaluation of groups in terms of Monomer Conversion degrees

Monomer Conversion Degree Values	Omnichroma	Vittra Unique	Charisma Diamond One	Zenchroma	
	Mean ± Sd	Mean ± Sd	Mean ± Sd	Mean ± Sd	^1^ *p*
Initial	0,78 ± 0,29	0,76 ± 0,12	0,74 ± 0,29	0,82 ± 0,1	0,504
^2^p	0,247	0,854	0,819	0,504	

^*1*^ *Kruskal Wallis Test* ^*2*^ *Friedman Test*

*p < 0.05

## Discussion

Due to dietary consumption, restorative materials within the oral cavity are subjected to various temperature and pH fluctuations. Within these dynamic conditions, the material’s surface properties play a pivotal role in determining the quality and performance of restorative procedures [13].

Despite significant dental composite resin restorative materials advancements, color stability remains a notable concern. Discolorations in composite resins can occur due to both intrinsic and extrinsic factors. Intrinsic discolorations may arise from factors such as the composition of the composite resin matrix (solubility/degradation) and inadequate polymerization. On the other hand, extrinsic discolorations are influenced by the material’s light-absorbing properties. Various parameters contribute to this discoloration, including water absorption, chemical reactions, dietary habits, smoking, poor oral hygiene, and the surface smoothness of the restoration [14].

Decreased oral cavity pH levels can lead to various alterations in restorative materials, such as increased surface roughness and discolorations. Surface roughness and irregularities make restorations more prone to dental plaque accumulation, potentially leading to gingival irritation and negatively impacting aesthetics [13]. Surface roughness of restorative materials in the oral cavity can create an environment conducive to bacterial adhesion, potentially resulting in secondary caries. The presence of pores on the material’s surface may also increase the likelihood of material discoloration.

The CIE L*a*b* system is widely adopted as an objective modality for assessing the colorimetric properties of dental composite resin restorative materials. This measurement system eliminates the inherent subjectivity in color perception and provides a standardized approach. Using a spectrophotometer allows for the reliable evaluation of changes in color attributes such as lightness (L*), red-green axis (a*), and yellow-blue axis (b*) [15].

This study exposed various monochromatic universal composite resin restorative materials to a red detox solution with a pH of 4.04. The duration and frequency of application were determined to simulate real-world detox application periods (21 days) and frequency (simulating three daily meals). The study assessed surface roughness and discolorations in restorative materials. While the statistical analysis did not reveal a significant difference in the surface roughness of the evaluated restorative materials, exposure to the detox solution increased the surface roughness.

The matrix content of composite resin restorative materials significantly influences their physical and mechanical properties. It is plausible to suggest that composite resins with a higher content of the hydrophobic monomer urethane dimethacrylate (UDMA) may exhibit reduced susceptibility to color distortion [16]. This phenomenon may explain why Vittra, among the monochromatic universal composite resin restorative materials evaluated in our study, exhibited the most stable discolorations.

UDMA, when compared to the hydrophilic bisphenol A glycidyl methacrylate (Bis-GMA), not only enhances the hydrolytic stability of composite resins but exhibits lower water absorption potential and higher resistance to discolorations. Water absorption in the literature has been attributed to the organic matrix’s Bis-GMA and triethylene glycol dimethacrylate (TEGDMA) monomers. TEGDMA’s small hydrophilic molecules have higher mobility in an aqueous environment [16, 17]. The movement of these molecules fills the voids occupied by pigment-carrying small water molecules. Therefore, ensuring that composite resin restorative materials are adequately polymerized is imperative. Complete polymerization, resulting in a higher degree of conversion from monomer to polymer, means fewer unreacted mobile monomers, leading to less water absorption and better color stability [18, 19]. Consequently, the specimens in this study were polymerized with a light-curing unit providing optimal light intensity.

In this study, all evaluated restorative materials exhibited discolorations after exposure to the red detox solution. However, Vittra, despite showing high discolorations in the initial and 7th day assessments, proved to be the most color-stable restorative material in the 21st day evaluation.

One crucial consideration in selecting dental restorative materials is their mechanical properties. Materials used in the restoration of teeth should be resistant to masticatory forces. Microhardness tests can be employed to assess these mechanical properties. Microhardness is associated with material composition and is influenced by aging, water absorption, and reactions occurring on the surface [20].

In recent years, the consumption of beverages such as cold-pressed fruit juices, similar to detox solutions obtained by mixing various fruits and vegetables, has increased [21]. Some acids found in the formulation of beverages, including acetic, propionic, and lactic acids, can decrease the microhardness values of composite resins [22]. Each material’s chemical composition and filler content differ, affecting their physical properties and hardness values [23]. In other words, factors such as filler particle size, filler ratio, monomer conversion degree, and the type of inorganic fillers can influence the microhardness of composite resin [24]. In this study, Omnichroma exhibited low microhardness values. This could be attributed to differences in the chemical components in Omnichroma, filler particle size and ratio variations, and structural differences in the supra-nano spherical fillers present in its composition. When examining the sub-surface microhardness values, Vittra Unique showed the lowest sub-surface microhardness values on the 21st day. Vittra Unique has the lowest filler content by weight among the tested materials. Researchers have reported a positive correlation between inorganic filler content and microhardness values [25]. In light of this information, Vittra Unique’s low microhardness values can be explained by its low filler content.

The degree of conversion (DC) is a parameter that indicates the polymerization degree of composite resins [26]. DC is crucial for the success of composite resin restorations, affecting mechanical properties, polymerization shrinkage, biocompatibility, solubility, color stability, and water absorption, among other characteristics. Internal and external factors influence the DC values of light-cured composite resins. Internal factors include the photoinitiator system, resin monomer type, quantity, and filler composition (filler particle size/type and quantity); external factors include polymerization time, polymerization mode, and the position of the polymerization device’s tip, as well as the light spectrum [9]. DC can be evaluated through various methods, including hardness assessment, optical microscopy, infrared spectroscopy, and Raman spectroscopy [27]. These methods can be used directly or indirectly to assess the degree of monomer conversion. An indirect method commonly used is microhardness measurement. This study used the ratio of sub-surface to surface microhardness values to evaluate the materials’ DC values [12]. This value significantly affects the mechanical and physical properties of the material [27, 28]. An ideal composite resin material should have a high degree of monomer conversion [26]. Therefore, composite resins aim to achieve low polymerization shrinkage and high conversion degrees by modifying organic and inorganic matrices [9]. In this study, no statistically significant difference was observed in the DC values among the materials.

## Conclusions

Within the limitations of this current study, it was concluded that: monochromatic universal composite resin immersion in the detox solution increased discoloration and surface roughness. Therefore, for individuals undergoing detox applications, it is advisable to consider restorations using these types of monochromatic universal composite resins, with more frequent post-restorative treatment follow-ups. Additionally, it was observed that the detox solution decreased the surface microhardness values of the mono-shade universal composite resin material containing supra-nano spherical fillers. Therefore, if planning a restoration with these materials, individuals should be advised to exercise caution regarding detox solution consumption, especially in the initial one-week period.

## Data Availability

No datasets were generated or analysed during the current study.

## References

[1] Akgül S, Gündoğdu C, Bala O (2022). Effects of storage time and restoration depth on instrumental color adjustment potential of universal resin composites. J Oral Sci.

[2] Gurgan S, Koc Vural U, Miletic I (2022). Comparison of mechanical and optical properties of a newly marketed universal composite resin with contemporary universal composite resins: an in vitro study. Microsc Res Tech.

[3] de Abreu JLB, Sampaio CS, Benalcazar Jalkh EB, Hirata R (2021). Analysis of the color matching of universal resin composites in anterior restorations. J Esthet Restor Dent.

[4] Oğlakçı B, Fazlıoğlu L, Tunç A, Özduman ZC, Dalkılıç E (2021). The effect of smoothies on the microhardness and color change of nano composite resin. Yeditepe J Dent.

[5] Kocaağaoğlu H, Aslan T, Gürbulak A, Albayrak H, Taşdemir Z, Gumus H (2017). Efficacy of polishing kits on the surface roughness and color stability of different composite resins. Niger J Clin Pract.

[6] Sarac D, Sarac YS, Kulunk S, Ural C, Kulunk T (2006). The effect of polishing techniques on the surface roughness and color change of composite resins. J Prosthet Dent.

[7] Miletic V, Stasic JN, Komlenic V, Petrovic R (2021). Multifactorial analysis of optical properties, sorption, and solubility of sculptable universal composites for enamel layering upon staining in colored beverages. J Esthet Restor Dent.

[8] Altıntaş S, Kılıç S, Gülnar A (2017). Hardness tests: surface hardness and measurement, surface roughness and measurement. Turkiye Klinikleri J Prosthodont-Special Top.

[9] Yılmaz Atalı P, Doğu Kaya B, Manav Özen A, Tarçın B, Şenol AA, Tüter Bayraktar E (2022). Assessment of micro-hardness, degree of conversion, and flexural strength for single-shade universal resin composites. Polym (Basel).

[10] Geha O, Inagaki LT, Favaro JC, González AHM, Guiraldo RD, Lopes MB (2021). Effect of chemical challenges on the properties of composite resins. Int J Dent.

[11] Bahadır HS, Aygör FA, Kelten ÖS (2024). Effect of detox drinks on color change and whiteness index of composite resins. Istanbul Kent Univ J Health Sci.

[12] Rouhollahi M, Mohammadibasir M, Talim S (2012). Comparative depth of cure among two light-cured core build-up composites by surface vickers hardness. J Dent (Tehran).

[13] Hengtrakool C, Kukiattrakoon B, Kedjarune-Leggat U (2011). Effect of naturally acidic agents on microhardness and surface micromorphology of restorative materials. Eur J Dent.

[14] Meshki R, Rashidi M (2022). Effect of natural and commercially produced juices on colour stability of microhybrid and nanohybrid composites. BDJ Open.

[15] Bahbishi N, Mzain W, Badeeb B, Nassar HM (2020). Color stability and micro-hardness of bulk-fill composite materials after exposure to common beverages. Materials.

[16] Sideridou ID, Achilias DS (2005). Elution study of unreacted Bis-GMA, TEGDMA, UDMA, and Bis‐EMA from light‐cured dental resins and resin composites using HPLC. J Biomed Mater Res B Appl Biomater.

[17] Chowdhury N, Wakasa K, Priyawan R, Yamaki M (1996). Matrix strengthening in new ternary bis-GMA/TEGDMA/urethane resin systems. J Mater Sci Lett.

[18] Garoushi S, Lassila L, Hatem M, Shembesh M, Baady L, Salim Z (2013). Influence of staining solutions and whitening procedures on discoloration of hybrid composite resins. Acta Odontol Scand.

[19] Korać S, Ajanović M, Džanković A, Konjhodžić A, Hasić-Branković L, Gavranović-Glamoč A (2022). Color stability of dental composites after immersion in beverages and performed whitening procedures. Acta Stomatol Croat.

[20] Ünal M, Candan M, İpek İ, Küçükoflaz M, Özer A (2021). Evaluation of the microhardness of different resin-based dental restorative materials treated with gastric acid: scanning electron microscopy-energy dispersive X-ray spectroscopy analysis. Microsc Res Tech.

[21] Yikilgan İ, Akgul S, Hazar A, Kedici Alp C, Baglar S, Bala O (2019). The effects of fresh detox juices on color stability and roughness of resin-based composites. J Prosthodont.

[22] Colombo M, Poggio C, Lasagna A, Chiesa M, Scribante A (2019). Vickers micro-hardness of new restorative CAD/CAM dental materials: evaluation and comparison after exposure to acidic drink. Mater (Basel).

[23] Ozkanoglu S, Akin EGG (2020). Evaluation of the effect of various beverages on the color stability and microhardness of restorative materials. Niger J Clin Pract.

[24] Shalaby HA, Metwally AA-H, Aboelenein AZ (2022). Water sorption, solubility, surface roughness and microhardness of omnichroma and essentia resin composite after thermocycling. Neuro Quantology.

[25] Chung KH (1990). The relationship between composition and properties of posterior resin composites. J Dent Res.

[26] Xu T, Li X, Wang H, Zheng G, Yu G, Wang H (2020). Polymerization shrinkage kinetics and degree of conversion of resin composites. J Oral Sci.

[27] Al Kheraif AA, Qasim SS, Ramakrishnaiah R, Ihtesham ur R (2013). Effect of different beverages on the color stability and degree of conversion of nano and microhybrid composites. Dent Mater J.

[28] Lin GSS, Abdul Ghani NRN, Ismail NH, Singbal KP, Yusuff NMM (2020). Polymerization shrinkage and degree of conversion of new zirconia-reinforced rice husk nanohybrid composite. Eur J Dent.

[29] Turgut S, Bagis B (2012). Color in dentistry and color measuring methods. J Dent Fac Atatürk Un.

